# Ebf Activates Expression of a Cholinergic Locus in a Multipolar Motor Ganglion Interneuron Subtype in *Ciona*

**DOI:** 10.3389/fnins.2021.784649

**Published:** 2021-12-17

**Authors:** Sydney Popsuj, Alberto Stolfi

**Affiliations:** School of Biological Sciences, Georgia Institute of Technology, Atlanta, GA, United States

**Keywords:** Ebf, COE, cholinergic, *Ciona*, tunicates, motor ganglion, acetylcholine

## Abstract

Conserved transcription factors termed “terminal selectors” regulate neuronal sub-type specification and differentiation through combinatorial transcriptional regulation of terminal differentiation genes. The unique combinations of terminal differentiation gene products in turn contribute to the functional identities of each neuron. One well-characterized terminal selector is COE (Collier/Olf/Ebf), which has been shown to activate cholinergic gene batteries in *C. elegans* motor neurons. However, its functions in other metazoans, particularly chordates, is less clear. Here we show that the sole COE ortholog in the non-vertebrate chordate *Ciona robusta*, Ebf, controls the expression of the cholinergic locus *VAChT/ChAT* in a single dorsal interneuron of the larval Motor Ganglion, which is presumed to be homologous to the vertebrate spinal cord. We propose that, while the function of Ebf as a regulator of cholinergic neuron identity conserved across bilaterians, its exact role may have diverged in different cholinergic neuron subtypes (e.g., interneurons vs. motor neurons) in chordate-specific motor circuits.

## Introduction

The myriad functions of nervous systems are made possible by the rich functional diversity of neuronal types and subtypes that are generated and connected to one another. Each terminally differentiated neuron in a neural circuit differs in their morphological, biochemical, and electrical properties. These properties in turn are often defined by coordinated gene expression changes regulated by transcription factors termed “terminal selectors” (Etchberger et al., 2007; Allan and Thor, 2015; Hobert and Kratsios, 2019). Terminal selectors can act alone or in combination to regulate the transcription of genes encoding rate-limiting effectors of terminal differentiation features. However, transcription factors that act as terminal selectors in one context might not function as such in other contexts.

Many terminal selectors have been shown to be evolutionarily conserved throughout animals, like Pou4/Brn3-family homeodomain factors expressed in various bilaterian and cnidarian neuron types (Finney et al., 1988; Eng et al., 2007; Serrano-Saiz et al., 2013, 2018; Zhang et al., 2014; Tournière et al., 2020). Another example comes from the COE (Collier/Olf/Ebf) family of transcription factors. A COE ortholog, UNC-3 in *C. elegans*, was shown to initiate and maintain the transcription of cholinergic genes, encoding essential components required for synthesis, transport, and reuptake of the major neurotransmitter acetylcholine (Kratsios et al., 2012) in motor neurons. Similarly, UNC-3 was found to control cholinergic gene expression in premotor interneurons as well, suggesting a broader function in regulating the development of cholinergic neurotransmission (Pereira et al., 2015). The COE ortholog Ebf was also found to be important for cholinergic motor neuron development in the invertebrate chordate *Ciona*, suggesting its role as a cholinergic motor neuron terminal selector is conserved from nematodes to chordates (Kratsios et al., 2012). However, subsequent studies on COE orthologs in vertebrates found a more nuanced role in motor neuron differentiation: different *EBF* paralogs are expressed in distinct spinal cord motor neurons innervating different axial muscles in mouse (Catela et al., 2019). Furthermore, Ebf2 is required for differentiation of a subset of motor neurons innervating epaxial (back) muscles, but not for their expression of cholinergic genes like *Vesicular acetylcholine transporter (VAChT)* (Catela et al., 2019). Thus, the regulation of cholinergic gene expression in vertebrate motor neurons may have shifted away from Ebf to another transcription factor, Islet (Cho et al., 2014; Catela et al., 2019).

Given the emerging differences between cholinergic gene regulation in vertebrate and invertebrate motor neurons, we decided to further investigate the role of Ebf in the regulation of cholinergic neuron identity in *Ciona.* With recent advances in tissue-specific CRISPR/Cas9-mediated mutagenesis (Stolfi et al., 2014) and the mapping of the *Ciona* larval connectome (Ryan et al., 2016), we were able to expand on this work with greater resolution than before. Based on these new tools and insights, we show here that Ebf activates cholinergic gene expression in a single neuron in the dorsal motor ganglion (MG) identified by the connectome as the Ascending Motor Ganglion Neuron 5 (AMG5) (Ryan et al., 2018; Kourakis et al., 2019). Although AMG5 is a cholinergic neuron situated in the major motor control center of the larva, it does not synapse directly onto muscles but rather onto other neurons of the MG, including primary motor neurons. We discuss these findings in the context of different scenarios for the evolution of cholinergic gene regulation in chordate motor circuits.

## Methods

*Ciona robusta* (*intestinalis* Type A) adults were collected in the San Diego, CA region (M-REP Consulting). Gametes were isolated and prepared for *in vitro* fertilization as previously described (Christiaen et al., 2009b). Dechorionated zygotes were transfected by electroporation as previously described (Christiaen et al., 2009a). All relevant sequences are described in Supplementary Sequence File 1. Embryos were raised at 20°C (unless otherwise stated) and fixed in MEMFA fixative (3.7% formaldehyde, 0.1 M MOPS pH7.4, 0.5 M NaCl, 1 mM EGTA, 2 mM MgSO4, 0.1% Triton-X100) for 15 min, rinsed once in PBS/0.4% Triton-X100/50 mM NH4Cl and once in PBS/0.1% Triton-X100, then finally mounted in 1X PBS/50% Glycerol/2% DABCO mounting solution. Images were acquired on inverted epifluorescence (Leica DMIL LED or DMi8) or scanning point confocal microscopes (Zeiss LSM 700). Confocal images were processed as maximum intensity Z projections using Zeiss LSM or ImageJ software. CRISPR/Cas9-mediated knockout of Ebf was performed using 30 μg *FOG* > *Cas9 (Gandhi et al., 2017)*, 40 μg *U6* > *Ebf.C (Gandhi et al., 2017) or U6* > *Control (Stolfi et al., 2014)*, 15 μg *FOG* > *H2B:mCherry (Rothbächer et al., 2007; Gline et al., 2009)*, and 90 μg *VAChT/ChAT -4315/-3886* + *bpFOG* > *Unc-76:GFP.* The Unc-76 tag has been previously used for efficient labeling of neurons especially their axons (Dynes and Ngai, 1998; Imai et al., 2009).

## Results

**FIGURE 1:**
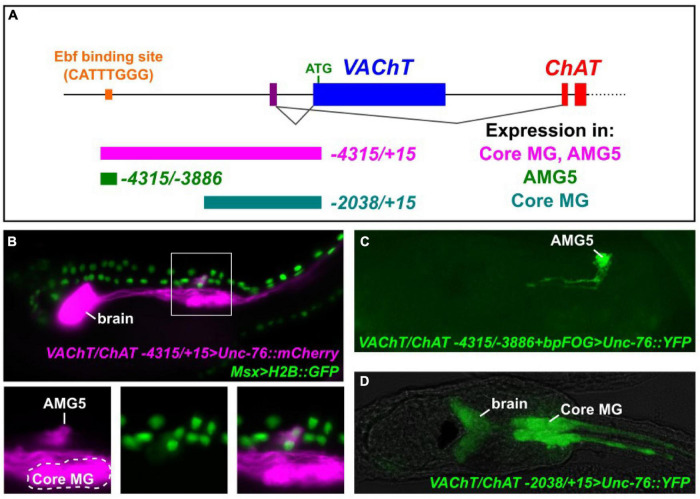
Expression of *VAChT/ChAT* cholinergic locus in the AMG5 neuron. **(A)** Cartoon schematic of the *VAChT/ChAT* locus encoding VAChT and ChAT from two mutually exclusive, alternatively spliced transcript variants sharing the same non-coding exon 1 (purple bar). Dotted line indicates additional exons encoding ChAT not shown. Colored bars underneath locus indicate *cis*-regulatory sequences tested, numbers reflect distance from the start codon (+ 1) of *VAChT*. Expression in “core” motor ganglion (MG) and Ascending Motor Ganglion neuron 5 (AMG5) was assessed qualitatively. An Ebf binding site previously reported (Kratsios et al., 2012) is situated -4111 bp upstream of *VAChT.* **(B)** Co-electroporation of *VAChT/ChAT* -*4315/* + *15* > *Unc-76:mCherry* and *Msx* > *H2B:GFP* revealed co-expression in AMG5 at 19 h post-fertilization (hpf) at 20°C, a cholinergic interneuron of the dorsal MG, derived from the dorsal cells of the neural tube. **(C)** A minimal fragment surrounding the Ebf binding site is sufficient to drive AMG5-specific reporter expression at 18 hpf, 20°C. Image is a confocal Z stack projection. **(D)** A smaller proximal fragment from *VAChT/ChAT (*-*2083/* + *15)* lacking the Ebf binding site is sufficient to drive expression in other cholinergic neurons including those of the core MG, but not in AMG5, at 19 hpf, 18°C.

We previously identified a predicted binding site for Ebf (CATTTGGG) approximately 4.1 kb upstream of the translation start codon of *Ciona VAChT*, based on the consensus sequence CCCNNGGG (Figure 1A; Kratsios et al., 2012). *VAChT* and *Choline acetyltransferase (ChAT)* form a cholinergic locus (henceforth referred to as *VAChT/ChAT*) in which alternative splicing of a shared transcript results in two distinct mRNAs coding for different effectors of cholinergic neurotransmission: VAChT (also known as Slc18a3) and ChAT. This peculiar arrangement is conserved from nematodes to chordates (Alfonso et al., 1993). A fluorescent reporter plasmid spanning -4315 bp upstream of the VAChT translation start that includes this predicted Ebf site was previously shown to drive expression in all cholinergic neurons of the larva (Figure 1B; Kratsios et al., 2012). This includes cholinergic neurons of the brain, MG, and in bipolar tail neurons.

While the “core” MG comprises bilateral pairs of neurons derived from the lateral rows of the neural tube which includes the two pairs of major primary, or lower motor neurons (Cole and Meinertzhagen, 2004; Ryan et al., 2016), a set of ascending motor ganglion (AMG) neurons is situated dorsal to the MG, relaying peripheral inputs to neurons of the core MG (Ryan et al., 2016, 2018). These have been shown to comprise a mix of cholinergic and GABAergic neurons and (previously called dvCNs and dvGNs, respectively) (Takamura et al., 2010). More recently, it was shown that AMG neurons 1–4, 6, and 7 are GABAergic, while AMG neuron 5 (AMG5) represents the sole cholinergic neuron in this cluster (Kourakis et al., 2019). Indeed, we found that the full-length (-4315/ + 15) *VAChT/ChAT* reporter labels a single cell just dorsal to the anterior half of the core MG, in the exact spot where we expect AMG5 to be (Figure 1B). Co-electroporation with the reporter *Msx* > *H2B:GFP*, which labels animal pole-derived lateral neural plate border cells that give rise to the dorsal row of the neural tube (Cole and Meinertzhagen, 2004; Russo et al., 2004; Stolfi et al., 2015; Figure 1B). This suggests the AMG5 neuron comes from one of the b8.19 blastomeres on either side of the embryo prior to neural tube closure (Cole and Meinertzhagen, 2004; Pasini et al., 2006). To our knowledge, this is the first evidence for the developmental origins of AMG5 from the Msx + dorsal row of the neural tube, although we could not ascertain whether this cell is invariantly derived from the left or right side of the embryo.

When we tested a smaller fragment surrounding the predicted Ebf site (-4315/-3886) in conjunction with a heterologous basal promoter (bpFOG) (Rothbächer et al., 2007), we found that this was sufficient to drive expression of fluorescent reporter solely in AMG5, but not any other cholinergic neuron (Figure 1C). This revealed the characteristic morphology of AMG5 as originally determined by the serial-section electron micrographs of the connectome study (Ryan et al., 2016, 2018; Ryan and Meinertzhagen, 2019), including unusual left and right ascending neurites (Figure 1C). Therefore we conclude that this region around the previously identified Ebf site corresponds to a *cis*-regulatory element that is sufficient to drive *VAChT/ChAT* in the cholinergic AMG5 neuron. In contrast, a shorter proximal fragment spanning -2083 bp upstream of *VAChT* (not encompassing the AMG5-specific element) was sufficient to drive expression in other cholinergic neurons, including the motor neurons and other “core” MG neurons (Figure 1D).

**FIGURE 2:**
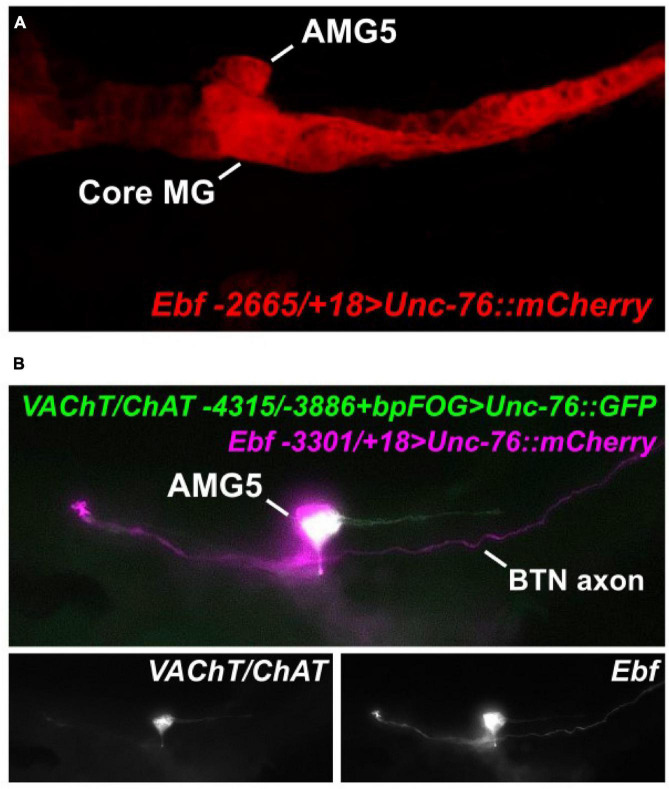
AMG5 co-expresses *VAChT/ChAT* and *Ebf.* **(A)** An *Ebf* reporter plasmid labels various CNS neurons including AMG5 at 18 h post-fertilization (hpf) at 20°C. Image is a confocal Z stack projection. **(B)** Co-electroporation of the *Ebf* reporter and the minimal AMG5-specific *VAChT/ChAT* reporter revealed co-expression in AMG5 at 17 hpf, 20°C. In this example, the larva shows mosaic incorporation of the plasmids only in the animal pole-derived lineages, which give rise to AMG5 and the Bipolar Tail Neuron (BTN) that also expresses Ebf (Stolfi et al., 2015), but not the core MG.

**FIGURE 3:**
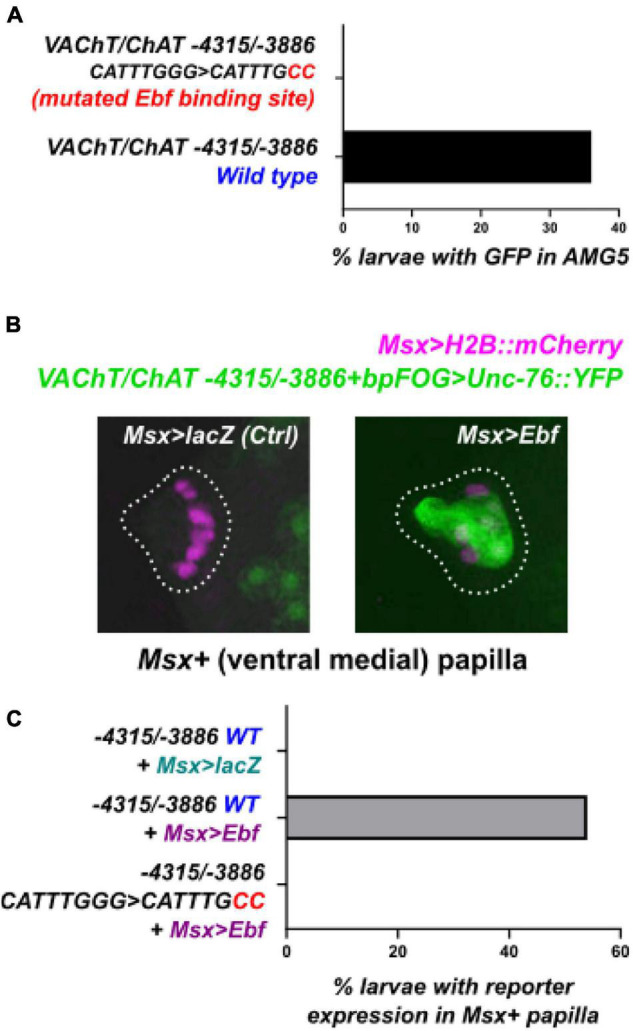
An Ebf binding site is necessary for *VAChT/ChAT* reporter activity. **(A)** Mutating the Ebf binding site in the minimal AMG5-specific *VAChT/ChAT cis*-regulatory element was sufficient to abolish its activity in AMG5. Mutated reporter *n* = 50, wild-type reporter *n* = 44. **(B)** The AMG5-specific *VAChT/ChAT* reporter can be ectopically activated in ventral papilla cells at 15.5 h post-fertilization (hpf) at 20°C, by overexpressing Ebf there (using *Msx* > *Ebf).* No expression is seen in the negative control electroporated with the neutral construct *Msx* > *lacZ* instead. Images are confocal Z stack projections. **(C)** Yet Ebf overexpression in the ventral papilla does not cause ectopic expression of the mutated *VAChT/ChAT* reporter, suggesting the Ebf binding site is indispensable for Ebf-mediated activation of this element. For each of the three conditions, *n* = 50.

Because of the predicted Ebf binding site in this AMG5-specific *VAChT/ChAT cis*-regulatory element (-4315/-3886), we hypothesized that Ebf might be directly activating *VAChT/ChAT* expression (and thus cholinergic identity) specifically in AMG5. Consistent with this hypothesis, we found that fluorescent protein expression driven by *Ebf cis*-regulatory sequences (Stolfi and Levine, 2011; Razy-Krajka et al., 2014) also labeled AMG5 and other cholinergic neurons, but not other surrounding (GABAergic) AMG neurons (Figure 2A). Co-expression with *VAChT/ChAT* reporter was confirmed by co-electroporation of *Ebf* and AMG5-specific *VAChT/ChAT* reporter plasmids (Figure 2B). Further, mutating the predicted Ebf site from CATTTGGG to CATTGCC in the context of the -4315/-3886 *cis-*regulatory element completely abolished reporter activity in AMG5 (Figure 3A). To test if Ebf can activate this AMG5-specific element, we overexpressed it in the ventral sensory papilla of the larva using the *Msx* promoter (Russo et al., 2004; Figure 3B). This strategy had previously been used to test ectopic expression of the full-length *VAChT/ChAT* reporter plasmid in response to Ebf overexpression (Kratsios et al., 2012). As expected, Ebf overexpression activated the *VAChT/ChAT* -4315/-3886 reporter in the papilla 54% of larvae, compared to 0% of larvae upon overexpression of a negative control *lacZ* sequence instead (Figures 3B,C). Furthermore, Ebf overexpression did not activate the expression of the *VAChT/ChAT* -4315/-3886 reporter carrying the mutated Ebf site (CATTTGGG to CATTGCC, Figure 3C). Taken together, these data suggest that Ebf directly activates an AMG5-specific *cis*-regulatory element for *VAChT/ChAT* expression.

**FIGURE 4:**
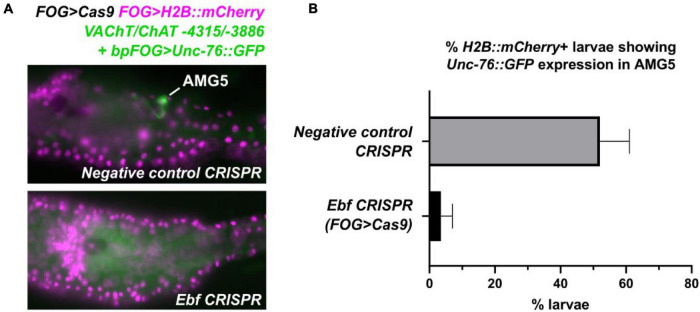
Tissue-specific CRISPR/Cas9-mediated knockout of *Ebf* abolishes *VAChT/ChAT* expression in AMG5. **(A)** F0 larvae co-electroporated with *FOG* > *Cas9, FOG* > *H2B:mCherry*, and the AMG5-specific *VAChT/ChAT* reporter (-*4315/*-*3886* + *bpFOG* > *Unc-76:GFP)*, and either a negative control guide RNA or an *Ebf*-targeting guide RNA (Gandhi et al., 2017) were scored for GFP signal in AMG5 at 17 h post-fertilization at 20°C. *FOG* > *Cas9* restricts CRISPR activity to the animal pole-derived cells from which AMG5 is born. **(B)** Scoring results of negative control and *Ebf* CRISPR larvae, averaged over two replicates. Negative control: replicate 1 *n* = 54, replicate 2 *n* = 49. *Ebf* CRISPR: replicate 1 *n* = 58, replicate 2 *n* = 43. Error bars indicate range between replicates. These results indicate that knocking out *Ebf* in the animal pole mostly abolishes *VAChT/ChAT* reporter expression in AMG5.

To verify that Ebf acts in *trans* to activate this minimal AMG5-specific *VAChT/ChAT cis*-regulatory element, we performed tissue-specific CRISPR/Cas9-mediated knockout of *Ebf* in *Ciona* embryos. We co-electroporated a highly efficient, previously validated single-chain guide RNA (sgRNA) vector targeting *Ebf* (Gandhi et al., 2017) together with *FOG* > *Cas9*, which drives Cas9 specifically in the animal pole-derived blastomeres at the 8-cell stage (Rothbächer et al., 2007; Gandhi et al., 2017). This combination was sufficient to abolish expression of *VAChT/ChAT* -*4315/*-*3886* reporter in AMG5 (Figure 4). Taking these manipulations in *cis* and *trans* together, we conclude that Ebf binds to and activates a distal *cis*-regulatory element of *VAChT/ChAT* that is activated only in AMG5, the sole cholinergic neuron in this region immediately dorsal to the “core” MG.

As for a possible earlier role for Ebf that could explain the loss of *VAChT/ChAT* reporter expression in AMG5, *Ebf* is not expressed zygotically in the lineage that gives rise to AMG5, before that cell is born. This is known thanks to detailed *in situ* hybridization screens (Imai et al., 2004, 2006, 2009). *Ebf* expression in the early embryo is non-existent until the late gastrula stage, when it comes up in the A9.32 pair of blastomeres and a pair of tail tip cells (none of which give rise to the AMG neurons). Therefore, we find it highly unlikely that CRISPR knockout of *Ebf* in the animal pole using *FOG* > *Cas9* is abrogating AMG5 specification prior to its birth.

## Discussion

We show here that *VAChT/ChAT* reporter gene expression in the dorsal motor ganglion region of *Ciona* requires the transcription factor Ebf. Since *Ebf* and *VAChT/ChAT* are co-expressed in a single neuron (recently identified as AMG5) in this region, we suspect that it may act as a classically defined terminal selector (Etchberger et al., 2007; Allan and Thor, 2015; Hobert and Kratsios, 2019) of cholinergic identity in this cell. The AMG neurons are a synaptically interconnected cluster of 7 ascending interneurons that receive synaptic connections from a variety of neurons processing diverse sensory inputs including light, gravity, touch, and possibly chemosensation. Their synaptic targets include neurons of the core MG and neurons of the brain (Ryan et al., 2016). It was recently shown that *VAChT/ChAT* is expressed in a single AMG neuron (AMG5), while the remaining 6 AMG neurons express *Vesicular GABA transporter (VGAT)* instead, indicating a GABAergic identity (Kourakis et al., 2019). AMG5 is a single multipolar neuron situated right on the midline surrounded by the remaining AMG neurons, with left and right axon branches projecting anteriorly. It receives heavy inputs from glutamatergic posterior apical trunk epidermal neurons (pATENs) and is presynaptic to prominent cholinergic neurons of the core MG including Motor Neuron 1 (MN1) and the Descending Decussating Neuron (ddN) (Ryan et al., 2016). Given its cholinergic identity and unique position and connectivity in the connectome, its function may be to trigger swimming behavior though cholinergic excitation of primary motor neurons, relaying an as-of-yet unidentified sensory stimulus transduced by the pATENs.

In *C. elegans*, the Ebf ortholog UNC-3 is a terminal selector for cholinergic neuron fate (Kratsios et al., 2012), suggesting a deep evolutionary history of this transcription factor as a determinant of cholinergic neuron fate. While the most abundant and prominent cholinergic neuron type in *C. elegans* is the motor neuron, UNC-3 does regulate cholinergic gene expression in cholinergic interneurons as well (Pereira et al., 2015). Similarly, expression of Ebf2 and other Ebf paralogs is seen in various neuronal precursors of the developing mammalian spinal cord, including those in the dorsal horn (Catela et al., 2019), where sparse expression of cholinergic genes has been observed (Mesnage et al., 2011). Given the role of dorsal horn interneurons in sensory integration, and the developmental origin of AMG neurons from the dorsal row of cells of the *Ciona* neural tube, AMG5 might be a homolog of these rare cholinergic interneurons of the mammalian spinal cord dorsal horn.

Although we previously used a dominant-repressor form of Ebf to suggest its role in specifying *Ciona* cholinergic motor neurons (Kratsios et al., 2012), we show here that a shorter *VAChT/ChAT cis*-regulatory fragment lacking a key Ebf binding site is sufficient to drive expression in motor neurons, but not AMG5. Therefore, it is likely that Ebf directly activates *VAChT/ChAT* in AMG5 but not necessarily in the neurons of the “core” MG that includes the primary motor neurons of the larva. This does not rule out a role for Ebf in regulating the activation and/or maintenance of other cholinergic effectors or more generic terminal differentiation genes in primary motor neurons. Additionally, there are cholinergic neurons, including primary motor neurons, in the post-metamorphic adults (Hozumi et al., 2015; Jokura et al., 2020), and it remains entirely unknown if these depend on Ebf for their specification and/or cholinergic fate.

Taken together, our results may help bridge the seemingly divergent roles of Ebf/UNC-3 in regulating motor neuron differentiation in *C. elegans* and mammals. While in *C. elegans* UNC-3 regulates cholinergic gene expression in primary motor neurons (Kratsios et al., 2012), Ebf factors in mouse regulate other aspects of motor neuron differentiation independently of their cholinergic identity (Catela et al., 2019). In the *Ciona* larva, we see a possible evolutionary intermediate between these two extremes. While Ebf directly regulates *VAChT/ChAT* expression in a cholinergic neuron of the motor ganglion that is immediately presynaptic to the primary motor neurons of the larva, its role in the primary motor neurons may reflect a more vertebrate-like function. Alternatively, there may be greater genetic redundancy in regulation of cholinergic gene expression in chordate motor neurons. A broader phylogenetic sampling may answer whether any of these (nematode, tunicate, vertebrate) closely resemble the ancestral condition in the last bilaterian common ancestor.

## Data Availability Statement

The raw data supporting the conclusions of this article will be made available by the authors, without undue reservation.

## Author Contributions

SP and AS designed, performed, and interpreted the experiments. AS wrote the manuscript, with edits and suggestions by SP. Both authors contributed to the article and approved the submitted version.

## Conflict of Interest

The authors declare that the research was conducted in the absence of any commercial or financial relationships that could be construed as a potential conflict of interest.

## Publisher’s Note

All claims expressed in this article are solely those of the authors and do not necessarily represent those of their affiliated organizations, or those of the publisher, the editors and the reviewers. Any product that may be evaluated in this article, or claim that may be made by its manufacturer, is not guaranteed or endorsed by the publisher.
