# Supplementary material for: Ebf Activates Expression of a Cholinergic Locus in a Multipolar Motor Ganglion Interneuron Subtype in Ciona
Source: Front Neurosci. 2021 Dec 17;15:784649. doi: 10.3389/fnins.2021.784649 (PMC8719597; doi:10.3389/fnins.2021.784649)
Supplement: Supplementary file 1 [file Data_Sheet_1.docx]

**Supplementary sequence file 1 - Popsuj and Stolfi 2021**

Longer *cis*-regulatory sequences are hypothetical sequences based on published genome assembly and may not reflect actual sequence cloned in plasmids, due to natural genetic variation.

VAChT/ChAT -4315/+15 Ebf site untranslated exonic START coding exonic

ccctactgtaacacagtaactggcggcttatatgccgttttctattaattacggggaagcatatcctaatggacaataaattaatatctgtgtcaacatgacaagtgactgaatatcgtaatgtaggcctaatatgaaacgtgttgagaattaaatcttcacagtggtaatgtgtatttcatgttacattgtgtactgtcttgtCATTTGGGaattgtgcagattgcagatcgtaatccaagagagaattatagacaagttttcttcaataatttctaattcttcattctctttacgagttcacttaccagttaattgacttttatatttcaaaatttcatttaaccagttttgcttaacctacggttagcttgcgctttcacacctctactttgaaataactgcgcgagctctggctagttttgcggttacctcgcagttttcacattaaatttaagtccagtcattgaataatcttatggaagaaattctgcatttcgtaatttttattgtatataacgttgttttaagcacacgttcaaaaatttaataaaagttacaataagttatttagcgatttgcacaacagctgaaattttaccaattcaaaatcacgcggggtaagatgagactgtttaaaatctttattagttttgaataataaatgaataatattagaaatacatgtaaacaacccgaaataatattgtacgctttgtaaatgaaaattaaacattctttccttgatccctgttatagtttttaacacgttgcatcccggttagcaaatgttccaccttaccccagtcccgtcttccccctacctactatcactcttacgattttcacatccccatgtgacgtataaccacataggcatagttccgatcgagcgatgaaacagtgatgttggttagcgtggggtaagatgagacacttaagaatatgttttctaatattaaaaaaataataaaattgactgttttttggtcatggcatgagtttgtattatcatagctttattaactgacaacatgaaatatataaaccacatataacgtatttatgggaattgatattgtttgaacgataattaataaaaattgtatatcctacagatgcaaataaacttataacaacagaaagttgacaatacttaataatgagattatatcggtatattaagtttaaactactcttgaggtttaaattattaaataacaaacaaaccgtttaaatttttgggtttgttttcttttattgttatcctatccttatgggtcaaccctctttaaagctccttttgtacgggtcgcgtctttacgtacatttctgtctctattagaagtttaactgaaaataattaaacataaatgactcgtttgcatgctaggtgtagcgaacacgcttattttcttccaattattgtaaatatgttcttaaatgtaagcgtattttaacaactgttgttttatcgcaatatcctgcatttttgttttaaatacttttaagtctttgctactataatcgaaaagacaaataaagtaattattaaaaggttaacaggttttatgtcaataatattctgcaaaacagtgtttttaattttatttttaatccaattcatactgctggctcctgccaattaataaaggtacattggcgtgggaaaagatgggacacctttagcacataatatctagatatcctgatcgcattttaaacaattaaacggtctaggagagtcgtgagggcaccactttatgattttttgaatgttctttgtttactaccaaatggaactatagaaagtcgaagaaaaaggtgtcctaccttaccccaccctacgatatatatccgtatttcttaaacggattttatgaaacaaaatcaaacagtacgaaatatttcagcctattttatttaactaaagtacttgattaatgatttaaactagcaaactacaaaattttgccaattttaatatgatttaccgagcaacttttattacttaaaaaaacggtaaatgcctttgccgcgcaatttgttagactgtgagaatcctgttgaatgttactaacattttgtttcacctcttatcaaataggtatcctacccagaaaagaagattttacaatattctattttaccccatgttccacgattccccactctgctatataatagtattttaaacaagtcgtagtttatagaggacttgagcgaaacttttattacgtcgtaaacctttggctaccatcatctgcctcaaaacaaaattaattaaagaaatgcgttagtgtatcctttgactcggaatcaatcaaatcaagcaaaaatcaatatgtgaaattaaccatttagaccttgtgtcattcccattgcggtgacttgtcctagttgtgcgtttttatcagcagattgatttaacagtcaccggaacaggcaagacaatatttcaaaccagccaatgttaatttcagacaaatgaagcaatctgaaaatcagaacaaatcaaaaaacataagttttggtttttaaagcatagaaaacgtaccgtattttttaatgtaattgttaaattttgttatttaatatagtagggtagggggagatgggacactttttcattctgttttctcgtcttggtagcaaacaaaaacattaaaagaattataaaaccgtatcctcgcgactcctacagaccgtttaaaacaggatatttggatattctgttctaaaggtgtcccatcttaccccacagtactatacactatacacattctgtaccgatatattttattgattaaatttgaagttgttaaacttaattacgattaaatttcggcaaattgaaaatgagccatgaattaatcaaaaattattcttgatcgttgttttgtaaaacataacttttttttgatttttttgggaggcgccctgtgttccacttattattatttgtttgttttgctatgtcaatactttaatataaaagaaattaatattggctgacatttcaatttaacgctaggcttatgttttgtttcgtgaataatctgcataaagaaaaaaagcagtatcgactctcctattgttgaatcactcttgcttcccttccattgtgcaggagatagtgacgcaatgaacattgattttaacgcttctagtcagttgggcccttgctcacaattgcctaaaatttgatcaattgtggattgaaaagttaatattctttcctgaattaacatgtctataggtaaggtattgagacggctcccatttaatttcttgtccgatctttataaaaaaatatttgttaaaggtacgtttaatttgaacatatagtgcagaagtcttcggaattttcatatagagttattttttagatactatatgcgacatattttcatagcccaaattaaattgttttataatttgtttggcttttacagaTTTTAATTTAACTTAGGTGTACGATTCAATAGAAAGATTTTAACCTGTAAAAATACGTGCAAGTTGTTTGGAAATACCTTTTTTGTTGGACAAAGTTGTATCAGgtaagttgatagcatgtatcttaaatctggcgtggtgtttatgtattggcttcatcatgaaatagtttgttttgtccttttacttgtcaattttatttaaattacatgagggtacaattcattataacgtacttcggtgaaaggttaatgttaacaaatgccgtccggtcttctgcgtatctttcgatttcgtattaattatgcatagaaagggtttaagtgcaatgctatttctgatgatgtgtgtgtaacaccaacgacctgatgacgaaaaacttgactgtttattaaataaaatagcgcaacaagacggcggaatttataaatggcatgttgctcgtaaacagcagcgcgtgcctgttttaactctggagaaacaagttatattcaactatttgttatctcacaaagcacggtattgcactcttatctaatgtactaatacaaatatatatatatatatatatatatatatctagctgttgtcgaggtaagatgaactgcagttaacaataaaaaccaacttcgttatacacaaattcaatataaagcgacagctcagtgttgtagagtaggtacccaactttcttaatctgcaaaaggcaaatacatgatttataatatgtaactcagcataacaacctgtttttgccttttgcagATTACATCTGAGGGCGGGTTGTGTGGGTCAGAAATTTATCAAAGGAAAATAAAGTTGATTGAAAGCAAATTTGTTTCTACTTTATTGTTCATCATGGACGTTTGTAGA

VAChT/ChAT -2038/+15 untranslated exonic START coding exonic

attacgtcgtaaacctttggctaccatcatctgcctcaaaacaaaattaattaaagaaatgcgttagtgtatcctttgactcggaatcaatcaaatcaagcaaaaatcaatatgtgaaattaaccatttagaccttgtgtcattcccattgcggtgacttgtcctagttgtgcgtttttatcagcagattgatttaacagtcaccggaacaggcaagacaatatttcaaaccagccaatgttaatttcagacaaatgaagcaatctgaaaatcagaacaaatcaaaaaacataagttttggtttttaaagcatagaaaacgtaccgtattttttaatgtaattgttaaattttgttatttaatatagtagggtagggggagatgggacactttttcattctgttttctcgtcttggtagcaaacaaaaacattaaaagaattataaaaccgtatcctcgcgactcctacagaccgtttaaaacaggatatttggatattctgttctaaaggtgtcccatcttaccccacagtactatacactatacacattctgtaccgatatattttattgattaaatttgaagttgttaaacttaattacgattaaatttcggcaaattgaaaatgagccatgaattaatcaaaaattattcttgatcgttgttttgtaaaacataacttttttttgatttttttgggaggcgccctgtgttccacttattattatttgtttgttttgctatgtcaatactttaatataaaagaaattaatattggctgacatttcaatttaacgctaggcttatgttttgtttcgtgaataatctgcataaagaaaaaaagcagtatcgactctcctattgttgaatcactcttgcttcccttccattgtgcaggagatagtgacgcaatgaacattgattttaacgcttctagtcagttgggcccttgctcacaattgcctaaaatttgatcaattgtggattgaaaagttaatattctttcctgaattaacatgtctataggtaaggtattgagacggctcccatttaatttcttgtccgatctttataaaaaaatatttgttaaaggtacgtttaatttgaacatatagtgcagaagtcttcggaattttcatatagagttattttttagatactatatgcgacatattttcatagcccaaattaaattgttttataatttgtttggcttttacagaTTTTAATTTAACTTAGGTGTACGATTCAATAGAAAGATTTTAACCTGTAAAAATACGTGCAAGTTGTTTGGAAATACCTTTTTTGTTGGACAAAGTTGTATCAGgtaagttgatagcatgtatcttaaatctggcgtggtgtttatgtattggcttcatcatgaaatagtttgttttgtccttttacttgtcaattttatttaaattacatgagggtacaattcattataacgtacttcggtgaaaggttaatgttaacaaatgccgtccggtcttctgcgtatctttcgatttcgtattaattatgcatagaaagggtttaagtgcaatgctatttctgatgatgtgtgtgtaacaccaacgacctgatgacgaaaaacttgactgtttattaaataaaatagcgcaacaagacggcggaatttataaatggcatgttgctcgtaaacagcagcgcgtgcctgttttaactctggagaaacaagttatattcaactatttgttatctcacaaagcacggtattgcactcttatctaatgtactaatacaaatatatatatatatatatatatatatatctagctgttgtcgaggtaagatgaactgcagttaacaataaaaaccaacttcgttatacacaaattcaatataaagcgacagctcagtgttgtagagtaggtacccaactttcttaatctgcaaaaggcaaatacatgatttataatatgtaactcagcataacaacctgtttttgccttttgcagATTACATCTGAGGGCGGGTTGTGTGGGTCAGAAATTTATCAAAGGAAAATAAAGTTGATTGAAAGCAAATTTGTTTCTACTTTATTGTTCATCATGGACGTTTGTAGA

VAChT/ChAT -4315/+3886 + bpFOG Ebf site basal promoter of FOG START

ccctactgtaacacagtaactggcggcttatatgccgttttctattaattacggggaagcatattctaatggacaataaattaatatctgtgtcaacatgacaagtgactgaatatcgtaatgtaggcctaatatgaaacgtgttaagaattaaatcttcacagtggtaatgtgtatttcatgttacattgtgtactgtcttgtCATTTGGGaattgtgcagattgcagatcgtaatccaagagagaattatagacaagttttcttcaataatatctaattcttcattctctttacgagttcacttaccagttaattgacttttatatttcaaaatttcatttaaccagttttgcttaacctacggttagcttgcgctttcacacctctactttgaaataactgcgcgagctctggctagttttgcggttTCTAGAGGATCCGGCAAAGCTTCGTGTATTGTACCGGCCCATTGTCAATCATGCAAACTTGATATTATATTGACAAGAGAAGAAGGCAGTTTAAATTAAAACTCTAAAGTAGAGAGACATTAATCTCAGCTGACAAGGCAGGTGGTCACAGTAAGTTCATTTAAATAGTTGGCCAACAATAGCCTTTCCAAGAAAGTATTTTTGTTCCAGGTCTATACAAAAATAACACACAACATG

VAChT/ChAT -4315/+3886 CATTTGGG>CATTTG**CC** + bpFOG Ebf site bpFOG START

ccctactgtaacacagtaactggcggcttatatgccgttttctattaattacggggaagcatattctaatggacaataaattaatatctgtgtcaacatgacaagtgactgaatatcgtaatgtaggcctaatatgaaacgtgttaagaattaaatcttcacagtggtaatgtgtatttcatgttacattgtgtactgtcttgtCATTTG**CC**aattgtgcagattgcagatcgtaatccaagagagaattatagacaagttttcttcaataatatctaattcttcattctctttacgagttcacttaccagttaattgacttttatatttcaaaatttcatttaaccagttttgcttaacctacggttagcttgcgctttcacacctctactttgaaataactgcgcgagctctggctagttttgcggttTCTAGAGGATCCGGCAAAGCTTCGTGTATTGTACCGGCCCATTGTCAATCATGCAAACTTGATATTATATTGACAAGAGAAGAAGGCAGTTTAAATTAAAACTCTAAAGTAGAGAGACATTAATCTCAGCTGACAAGGCAGGTGGTCACAGTAAGTTCATTTAAATAGTTGGCCAACAATAGCCTTTCCAAGAAAGTATTTTTGTTCCAGGTCTATACAAAAATAACACACAACATG

Msx -2304/-74 exon 1

ctacgcattgatgtcgcaatctccccaagtttcccaccacctatatgaaagctacgaaggcggaaagatttcggcataataacctattgttgacgtcataatcgaccgataaataagcggcgaagatcgcgtcctctacaataggaggtggaacactcataaaaaatcacagcggtaaagccgctaccacacgctgctaacatttattaaggagacatgctacactcgacgtcataatgcgcgtgacgactatattgtgacgtcacagagagataaataggatatttattgcgcggtagaatagaacgacgagcaataaaatgaaatgaattttggaaatggtaaattaatttattttataagtcgtctaaactttatccatttccgtataaaacacgacttttaccaactgttaatttttattaacttaccacataaagtagtttattgggccgctctgaacgaatggaaatcaccgtactctattaagttgatcgaccttttacatttcaatatgttaaaaatcgtacattaaaaaaagatatagtaggatggggggatgggatacgttttcattctattttcttgtccaatttggtagtaaaaaaggaactttcaaagatttataaaaccgcatccttacgactttcatagacggatggtaattgtttaaaacacgaacagtatattaagataatattttctaatggcgtcccgtcttcccccgccctactatattaaaaagaggcaatatgaaccaatacttaagattttagttggtgcacatcaattactgtttttataagtccatgcagaccgatgtccatttaccagtgattaaacagggcaataaaattacgaagaatcgccttgcgtcgctgcaaacatatttattaggcattagcaagtgtgacgtaataatgcctcttgttcgacgtgacggcgagcgcttgcttttgaaacaccgcgacgtcgacgaagacggtgtcgtaacttaacaatagtctggggcctatgatgtcataatgcccatttggtgcactcgcgtgctgtcgcccaaaatcgcaattaactcgatgacactggcagcatatgacgtcataaaatggttgccagggtcccggaccgagacagtaattcacttttatgacccgaatatctaatgggacccatgatgtacacttagaagcacgcagcaactgattattaatgttgagtgtgttacggatgactgttaatttcacaaacaaacaaaaaaacagagttcgttggcctttagatcactattagaaacaacagtaattggtgggaacttgtttcggagcgagccaaataaaaaagctaaaacaaacataacagtaaagcttaattaaaataataagcgttgttaataaatattagcatatagaccaaacaccaaattgtgtgcaatcagtttttgcatctttaaaactattaattttttatgttcgccgtgcgaatttttatttgaatgattctattaataaagcctgaaatcctagcaatcattttgagcttccacatttgatattaaaattctcgtatttgttgttatttattaatgattttgagtgacttttttagactcagtttttgttttgtttttcaattattcaagttcggatgtgtggagatacgggattaatacaatgaatgttcaattaaaaacacaaaagttttaatttgaattaatgtcggcgctgcagacgagccgagagcttgtcggatttaaaatgtaatcctccaattttgtctggcactgacggtgtagatgcaatctgaaaatggcgactgagcaggatcgggtcgcggctggagctccggcgggctcgggacgctcataattccgccggtaatccccgtaacgtcgatgaaagcgaacgcgccgacaaaagtgacgaagattaagtgtaacaacatggtttaaacaaacagactggagcagcggagacgagagagagagggaacgtaatcgccgcggccgaagcgtgaccaagtcgttgtattgttataatagaaaatcgttttattatgatttaatgaacgcgcgctggcgactgaaggatgctgtataattgggggtaatggggaaatatcgggagaaaaaatatggcgatctgcgggagaggatagtcgcttacataatctctggagtttAGTTGAAGCAGTTCATATTGAGAGAAGCATCCCTCAGACGAGTAGTTCGTAAA

Ebf –2665/+18 exon 1a START

gtccattatttgtctgcagggaaaagtctctggcaaagtttaatgaattcttccgggaaataagagcggcagcgactttattcgaatttcaaatttccatgatattcgataccccacactccaccctataacggtgacctggtttaggaaatatgtgacgttgcttcacgacaattttagcagctatataagttaccattgcaaaaaagatcagtctttttatttatcacaatttttaccagatttttaaatattttttagaaataacttaaattttgatcaaactggattaaaattgcaaattttacatttttgtgataaatattaatctttttagtcgccatggcttaaaaatgtcatttgtttagtccgctgtattcaattttaccgtttgataaatcaaacggttaacttgcgcataaaaccaatttctcatatggatattactccgcgcgagttctaacaacgcgttctcttcgctggccaagatcgatactccgtgacgtcacaatcacatgcgcgcgtctgaaatatggtcgacagagttgcaacacgcaacccttttgtttgggttaatttccctctttgttttttaagtccgatcgcgtcgccacatctgttcgacgaatcttcgttctgtcttagcaactttgcggctctgtccattgtgaagtcgtaaacgaggcattgtcgtcactgctgtctcgcctacgtcacaaagcagtgcacagtgacgtcacagagacgaggtcgcgtgccttcgagttggaaaattcaaagcattgcactttttgccgaatgttaatttttacgacggaagaagttaggacaagcataaaataacaaataaataacccataaaaaacgtaaaaacaaacaagagaacggtattcaaaattagaatttcgaaaaaaatgttaaaaaataatacttagagtcgctgaacaatttaagccgacaaaggccacaaaactgcctaaaatttataaaaaaaatgtaaaattattttgttttttttaaactacagttatcacctttaaaacaaacaaattagcaaacgttgtaattacttcacaactttcttgcgacgctaaaaggcggcgaattttattgctattgtgacgtcacaagcgctctcgtcacgcccggatacgattagaacaacgaaggattgtttgtttttaattatttctctgtttaatcatttgatttagcgcggcacaaattttgttttatataaaatgtatccattttatctctgcgcgtttttgtactattttttgaaaaatgtttgttaaccttttgaaaatcgcgaaaccaacgaaattatttccaaaggctgtacaattctttttcgttaggttacgtgtttaagtataggcccaagttttaaatggcggacagagtttcgtattttgatatttgaatttttttgaaatcttgaaaaaaaataattgtacgttttacatagaataactaaccaaaatatctgaagcaaaaattacgtacaattttttaaaatgtaatttactttctagcttttaatttttgtgcttttctcaatatgtgtgccatattttaaaaacgtaaatttgcctgttgttaagcggaggaaaaagtaatctgcgtgaatgcgaaaaacacgattttggaatcagcgccggcaatggtgtttgtaaataggggtggcatacgcgtttcgtagcgaaagagagaatgaggcgaaagtcgacagatgcacgctccgatttatgagacaggaaccagtcgcgagggcacggaggaaaaagaaccttactccaagacatgcgccgccttttttctttctgtcctagtcaggaatactagagtatagaaggccacgcgtcgtggagtttaaaaccagcaagccagtgtctcaacggacacatcaatacagagacttctctcagtggacaactcggacgattcgccactaacttggtggattcgtcccggacgacccatgggccgggtcccagcgcgctagttggccaccatacagtgtagaatcagctagatcgtctctgcggatttcgcaaatagattgagttggagatagcttcccgaccgggatttcgactaatttgcaatgttagttattaatcaaggtgacagtcaggagttaaagttaatttaccttttgaaagggcaaaaagattttcgaagtaaattgattccgttaattgtaactcttaaacgcaaaccgataaacgacgccattttgcttttcattgagaaactaaccatttaggcattctataattaaaattaaatgtttttaaaatctgtaactatctgtaataaactagaattatttatttcagtttaaatttttatttaaaacaataattttattatttcctttattcatcctaatgatattggtatagagaaaacgatgtttttatttccataaaattttatttcagaaaagtcattttgttcaatttaaaaaattcctttattctaaaaaaatgcccaaaacaagttctttatttcttaaaaattatcctaaataaaaaaatccacgttttaataaaatgtataaaattgaaaactataaaaagatgacttatttttttaccctaacgtgatttttcaccagATACCTTAAGTGTTATTTTATTTGTAAGTAATATCCAAATGGCAACAATCGCGGGA

Ebf -3301/+18 exon 1a START

aattaagcgtcttttcgaatcgtgagtatacggttatatacgacgggataaaaaacacaactaaaacaaggaccatattaccccaacctactattgtgtttcgttttactcggtaccactatcttttaaattattatgtagtacggtggggggagatgggagaacttttcattcccttttttgtcccatttggtggtaaacaaagaacattcaaataattattaaaccgtatccccacggctctaatagactgttgttaattgtttgaaacaagatcaggatctttgaatatttgtgctaaatgtgtcccatctttcccaccctacatatatagtacaaaatacagccagtatctattaacttgtatgggcacttcggtcgctgaggaagtcatcattctacaccagacacttttaaacaaacaagacgtgcgcggtaacaggttcacccgtccttattggttcaatagacctcattatgacgtgacaatcgttcccgtaggacgtttttgtgccaggaagcaaacctttcggtgggttcgaatccctaggcggtgtgctaaagtggcggctggctaattaaaataactaaaagtcgggccacgtctttgttttttcaactcgatataatttctgtccattatttgtctgcagggaaaagtctctggcaaagtttaatgaattcttccgggaaataagagcggcagcgactttattcgaatttcaaatttccatgatattcgataccccacactccaccctataacggtgacctggtttaggaaatatgtgacgttgcttcacgacaattttagcagctatataagttaccattgcaaaaaagatcagtctttttatttatcacaatttttaccagatttttaaatattttttagaaataacttaaattttgatcaaactggattaaaattgcaaattttacatttttgtgataaatattaatctttttagtcgccatggcttaaaaatgtcatttgtttagtccgctgtattcaattttaccgtttgataaatcaaacggttaacttgcgcataaaaccaatttctcatatggatattactccgcgcgagttctaacaacgcgttctcttcgctggccaagatcgatactccgtgacgtcacaatcacatgcgcgcgtctgaaatatggtcgacagagttgcaacacgcaacccttttgtttgggttaatttccctctttgttttttaagtccgatcgcgtcgccacatctgttcgacgaatcttcgttctgtcttagcaactttgcggctctgtccattgtgaagtcgtaaacgaggcattgtcgtcactgctgtctcgcctacgtcacaaagcagtgcacagtgacgtcacagagacgaggtcgcgtgccttcgagttggaaaattcaaagcattgcactttttgccgaatgttaatttttacgacggaagaagttaggacaagcataaaataacaaataaataacccataaaaaacgtaaaaacaaacaagagaacggtattcaaaattagaatttcgaaaaaaatgttaaaaaataatacttagagtcgctgaacaatttaagccgacaaaggccacaaaactgcctaaaatttataaaaaaaatgtaaaattattttgttttttttaaactacagttatcacctttaaaacaaacaaattagcaaacgttgtaattacttcacaactttcttgcgacgctaaaaggcggcgaattttattgctattgtgacgtcacaagcgctctcgtcacgcccggatacgattagaacaacgaaggattgtttgtttttaattatttctctgtttaatcatttgatttagcgcggcacaaattttgttttatataaaatgtatccattttatctctgcgcgtttttgtactattttttgaaaaatgtttgttaaccttttgaaaatcgcgaaaccaacgaaattatttccaaaggctgtacaattctttttcgttaggttacgtgtttaagtataggcccaagttttaaatggcggacagagtttcgtattttgatatttgaatttttttgaaatcttgaaaaaaaataattgtacgttttacatagaataactaaccaaaatatctgaagcaaaaattacgtacaattttttaaaatgtaatttactttctagcttttaatttttgtgcttttctcaatatgtgtgccatattttaaaaacgtaaatttgcctgttgttaagcggaggaaaaagtaatctgcgtgaatgcgaaaaacacgattttggaatcagcgccggcaatggtgtttgtaaataggggtggcatacgcgtttcgtagcgaaagagagaatgaggcgaaagtcgacagatgcacgctccgatttatgagacaggaaccagtcgcgagggcacggaggaaaaagaaccttactccaagacatgcgccgccttttttctttctgtcctagtcaggaatactagagtatagaaggccacgcgtcgtggagtttaaaaccagcaagccagtgtctcaacggacacatcaatacagagacttctctcagtggacaactcggacgattcgccactaacttggtggattcgtcccggacgacccatgggccgggtcccagcgcgctagttggccaccatacagtgtagaatcagctagatcgtctctgcggatttcgcaaatagattgagttggagataggttcccgaccgggatttcgactaatttgcaatgttagttattaatcaaggtgacagtcaggagttaaagttaatttaccttttgaaagggcaaaaagattttcgaagtaaattgattccgttaattgtaactcttaaacgcaaaccgataaacgacgccattttgcttttcattgagaaactaaccatttaggcattctataattaaaattaaatgtttttaaaatctgtaactatctgtaataaactagaattatttatttcagtttaaatttttatttaaaacaataattttattatttcctttattcatcctattgatattggtatagagaaaacgatgtttttatttccaaaaaattttgtttcataaaagtcattttgttcaatttaaaaaaataccttattctaaaaaatgcccaaaacaagttctttatttcttaaaaactatcctaaataaaaaaaaactccacgttttaataaaacctataaaattaaaaactataaaaagatgacttatttttttaccctaacgtgatttttcaccagATACCTTAAGTGTTATTTTATTTGTAAGTAATATCCAAATGGCAACAATCGCGGGA

Ebf coding sequence (transcript variant 1: KH.L24.10.v1.A.SL1-1)

atggcaacaatggcgggacctcagttatcggggccagcagtgagaggatggatgcaaacaacgttagtggaaccgatgccaaacggcaatgttggtctacatcgggcacatttcgaaaagcagccgccaaacaaccttagaaaaagcaactttttccacttcgtgcttgccctgtacgacagacaagggcagccggtggaaattgagcgaagtgctttcgtaggatttgtggaaaacgaaaccgagatcgccggcgaaaaaacaaacaacgggatccagtacagactccaattgctttaccatagcggcgtacggacagaacaagatgtattcgtccgtcttatcgactctgcaaccaaacagtcaattacgtacgaaggacaggataaaaacccagaaatgcgacgggttttgctcactcatgagatcatgtgcagccgctgctgtgacaagaagagttgcgggaacagaaacgaaactccatccgacccagtggtgattgacagatacttcttaaaattctttctcaagtgcaaccaaaactgtttaaagaatgctggaaatccaagagacatgcgaagatttcaggttgtggtctcaacaactgtgcatgttgacggtcatgttttagctgtgtctgacaacatgttcgtgcacaacaactcgaaacatggacgtcgagcgagaagggtcgacccatcagaagcttcgccaactatcaaagcaatcaacccagctgagggttggacaacaggtggagcaacagttgttatagttggagaaaatttctttgacgggcttcaagttgtgtttggttcaatggttgtatggagtgagttgatcacccaacacgcaatcagagtacagactccaccgcgccatctaccgggcgtggtagaagtgactctgtcttacaaaaataaacaattctgcagtggtgctccaggacgctttgtatacacagcgctcaacgaaccgacacttgattatggattccagcgattactgaagactgtgccaagacacccaggcgaccctgagaggctaccaaaggagattattctaaaacgagctgctgacgtcatggaagcggtgatcagtcggcagtacgccccgccaagccaaatgcccccgtcagccgggatcacgcctccggcgccccatctagcCgcGgcaccgtgcgcaccacctggcagtttcgttccgcaatctgctagcgctgccatggccgtggcaatgaacggttacgctgctgcagcggtttcttcacaatttggcggaacacccgatcggttcgacaccggaagtgattcaggttactcacgaggtaacagtgtttctccccgcaacggatattccccccagacgacaccgcatagcttgaacagtggctcaattggtagcatggtggggctcacaactgtaggcgcagttccggccccagccccctaccactgcgcaccatcctttaacagttattccagcgcttcaggtcccacattcacgaacatgaacaacacgtctcctggccttttttctggatctgggataattccaccttcgccccacaacggtatgaacccactcccgtcttcggggaccacaccgggaatttttagtttttcacctgcaaacatgatatcagccgcaaagcagaagagcgcatttgcccctgtacatcgtccacacaactcgcccagtcctctagcgccatctaacggaaatatcgcactaaacggctatagctag

FOG>Cas9 plasmid FOG -1982/-1 NLS::Cas9::NLS

ggcgcgccGCAACTATTGTAACACCACACGGGGGCGCAGTAGAGCCAGAATCTTAGTAAATGCGGTTTGATTGTAAAGTTTTTAACAATCTCTCGCTTTGTATATTCTAGAATGGGGAAAGATGGGACCCTTAAGCACTCATTGCCCAATATTTCCAAACCCAAAATCTACGATGGTTTTGTGTGGTACCACGAATTAGTACTATCATATACACTTCAAAGAAATATATTAAACCATACGAGAAGTAATTTATCTATTCGCACAACAGGTGTAACTTTGGAAATTCGAACACAGGCGAGCTAGTTTTATGACACGTAAACTTTATATATTTCTTTTCTAATTTAAGGAATATTGACCACTGCCCATTTTTCTTGTTTTAATATCCCAGTTTTTAAAATGGCCTGAAATATTTCAGCTTATTTTATTAAATTACGATACTGGATTGATGATTCAAATTAACAAACTACCAAAAAATGTTGACAGTTTTAATATGATTTACCGAGCACCACGTGTAACAGCATCATACAAATTTAATTGATTTTGTATTACTTACTTTATAATTTAAACATACGGTATTTAGTAAACAAAGAACATTCAAAGAATTAGAAAACCGTATTCCCATGACTTTCATAGACCGTTGTTGATTGTTTAAAACATGATCATAATATGTAGGTATTATATGTGCCAAAGGTGTCCCGTTTTCCCCCACCCATACTATATATATTATAAAAAAAAATGGCGTTATAAAAAAACTATACCCTGCATGTATTTATAGATTTGTTAAACGTATGATCTCGTGTTATGATCTATCCGTATAGTGGACCAGGAATCTTTGGGTTGTGCATATTTAACTGAGAAATCGACAAGCAATTAAACGCGGCAGTATACGACATGAACAATATAATTGGCGCATTATAATTAGATTGTATACCACGCACTACCTAAAAAGTTAAGCTATAACTGACGTCAGGTATTATTTATTGGTATCCGGCAACACTAATAAATGAAATTTTGTCAAAATGTAAGCCCTCGTATCTATTTAAATTTTGACGCTAAATAATCCATTTATGCAGTATAATACCAAACATACAAAATGTTTCAGTTTGTATCACTTTATTATGCATCACGTTTTTGGGATAAATACACATCAGGATTTAACAAAATATGTTAAATACAATATGGCGGTATTGCTTTCACTTAAAAGTGTTAACAATTAAGATGGGAAAAATAAAACAGTAAATATATTCTTTAAATCGAACTTTTGTTGTAAATCTTCTATTTTAATAATTTATTCATGTTTAAAACGATGTTTGTTTCGGCGTCATGCTGCTCGTTAGGTATAACTTCACTCGGGACATTTTAGTATCGAGTTGCTCAAAATTGTTAGGGTTGTCAACTGAATAGCGATGTTTTCGATTGAGTTAATATTTCTATACATGATGGCATCAGCTAGTAAGATAGAAAACAACCTTGTTATTACTCGTGCTGTAATAATATAATTCATGAAAAACAAACATGGTCTTATCAAATCTATTCCGAAGACAAACATACGGCTGATAGAATTCAGTCAGCGCGGTTTGGGCGACATCACTCAGAGCTCTAGCTCTTATCTTGCTTATCGAGCAAGCGATAAAACAAGCAGTAACGAGAAAACAAGAGAAAGAAGGCTAGCTTCCTGGAGAAGACCAAGATAAAGTATCTCAAAATTCAGGAAACGGTCCAAGACCGAAGCTCCAAAGCTCTTGTGTTCAGTTAAACTCTGATAGTGAATAAGCTTCGTGTATTGTACCGACCCATTGTCAATCATGCAAACTTGATATTATATTGACAAGAGAAGAAGGCAGTTTAAATTAAAACTCTAAAGTAGAGAGACATTAATCTCAGCTGACAAGGCAGGTGGTCACAGTAAGTTCATTTAAATAGTTGGCCAACAACAGCTTTTCCAAGAAAGTATTTTTGTTTCAGGTCTATACAAAAATAACACACATAgcggccgcaaccATGGCTAGCCCCAAAAAGAAGAGGAAAGTGGACAAGAAGTATTCTATCGGACTGGACATCGGGACTAATAGCGTCGGGTGGGCCGTGATCACTGACGAGTACAAGGTGCCCTCTAAGAAGTTCAAGGTGCTCGGGAACACCGACCGGCATTCCATCAAGAAAAATCTGATCGGAGCTCTCCTCTTTGATTCAGGGGAGACCGCTGAAGCAACCCGCCTCAAGCGGACTGCTAGACGGCGGTACACCAGGAGGAAGAACCGGATTTGTTACCTTCAAGAGATATTCTCCAACGAAATGGCAAAGGTCGACGACAGCTTCTTCCATAGGCTGGAAGAATCATTCCTCGTGGAAGAGGATAAGAAGCATGAACGGCATCCCATCTTCGGTAATATCGTCGACGAGGTGGCCTATCACGAGAAATACCCAACCATCTACCATCTTCGCAAAAAGCTGGTGGACTCAACCGACAAGGCAGACCTCCGGCTTATCTACCTGGCCCTGGCCCACATGATCAAGTTCAGAGGCCACTTCCTGATCGAGGGCGACCTCAATCCTGACAATAGCGATGTGGATAAACTGTTCATCCAGCTGGTGCAGACTTACAACCAGCTCTTTGAAGAGAACCCCATCAATGCAAGCGGAGTCGATGCCAAGGCCATTCTGTCAGCCCGGCTGTCAAAGAGCCGCAGACTTGAGAATCTTATCGCTCAGCTGCCGGGTGAAAAGAAAAATGGACTGTTCGGGAACCTGATTGCTCTTTCACTTGGGCTGACTCCCAATTTCAAGTCTAATTTCGACCTGGCAGAGGATGCCAAGCTGCAACTGTCCAAGGACACCTATGATGACGATCTCGACAACCTCCTGGCCCAGATCGGTGACCAATACGCCGACCTTTTCCTTGCTGCTAAGAATCTTTCTGACGCCATCCTGCTGTCTGACATTCTCCGCGTGAACACTGAAATCACCAAGGCCCCTCTTTCAGCTTCAATGATTAAGCGGTATGATGAGCACCACCAGGACCTGACCCTGCTTAAGGCACTCGTCCGGCAGCAGCTTCCGGAGAAGTACAAGGAAATCTTCTTTGACCAGTCAAAGAATGGATACGCCGGCTACATCGACGGAGGTGCCTCCCAAGAGGAATTTTATAAGTTTATCAAACCTATCCTTGAGAAGATGGACGGCACCGAAGAGCTCCTCGTGAAACTGAATCGGGAGGATCTGCTGCGGAAGCAGCGCACTTTCGACAATGGGAGCATTCCCCACCAGATCCATCTTGGGGAGCTTCACGCCATCCTTCGGCGCCAAGAGGACTTCTACCCCTTTCTTAAGGACAACAGGGAGAAGATTGAGAAAATTCTCACTTTCCGCATCCCCTACTACGTGGGACCCCTCGCCAGAGGAAATAGCCGGTTTGCTTGGATGACCAGAAAGTCAGAAGAAACTATCACTCCCTGGAACTTCGAAGAGGTGGTGGACAAGGGAGCCAGCGCTCAGTCATTCATCGAACGGATGACTAACTTCGATAAGAACCTCCCCAATGAGAAGGTCCTGCCGAAACATTCCCTGCTCTACGAGTACTTTACCGTGTACAACGAGCTGACCAAGGTGAAATATGTCACCGAAGGGATGAGGAAGCCCGCATTCCTGTCAGGCGAACAAAAGAAGGCAATTGTGGACCTTCTGTTCAAGACCAATAGAAAGGTGACCGTGAAGCAGCTGAAGGAGGACTATTTCAAGAAAATTGAATGCTTCGACTCTGTGGAGATTAGCGGGGTCGAAGATCGGTTCAACGCAAGCCTGGGTACCTACCATGATCTGCTTAAGATCATCAAGGACAAGGATTTTCTGGACAATGAGGAGAACGAGGACATCCTTGAGGACATTGTCCTGACTCTCACTCTGTTCGAGGACCGGGAAATGATCGAGGAGAGGCTTAAGACCTACGCCCATCTGTTCGACGATAAAGTGATGAAGCAACTTAAACGGAGAAGATATACCGGATGGGGACGCCTTAGCCGCAAACTCATCAACGGAATCCGGGACAAACAGAGCGGAAAGACCATTCTTGATTTCCTTAAGAGCGACGGATTCGCTAATCGCAACTTCATGCAACTTATCCATGATGATTCCCTGACCTTTAAGGAGGACATCCAGAAGGCCCAAGTGTCTGGACAAGGTGACTCACTGCACGAGCATATCGCAAATCTGGCTGGTTCACCCGCTATTAAGAAGGGTATTCTCCAGACCGTGAAAGTCGTGGACGAGCTGGTCAAGGTGATGGGTCGCCATAAACCAGAGAACATTGTCATCGAGATGGCCAGGGAAAACCAGACTACCCAGAAGGGACAGAAGAACAGCAGGGAGCGGATGAAAAGAATTGAGGAAGGGATTAAGGAGCTCGGGTCACAGATCCTTAAAGAGCACCCGGTGGAAAACACCCAGCTTCAGAATGAGAAGCTCTATCTGTACTACCTTCAAAATGGACGCGATATGTATGTGGACCAAGAGCTTGATATCAACAGGCTCTCAGACTACGACGTGGACCACATCGTCCCTCAGAGCTTCCTCAAAGACGACTCAATTGACAATAAGGTGCTGACTCGCTCAGACAAGAACCGGGGAAAGTCAGATAACGTGCCCTCAGAGGAAGTCGTGAAAAAGATGAAGAACTATTGGCGCCAGCTTCTGAACGCAAAGCTGATCACTCAGCGGAAGTTCGACAATCTCACTAAGGCTGAGAGGGGCGGACTGAGCGAACTGGACAAAGCAGGATTCATTAAACGGCAACTTGTGGAGACTCGGCAGATTACTAAACATGTCGCCCAAATCCTTGACTCACGCATGAATACCAAGTACGACGAAAACGACAAACTTATCCGCGAGGTGAAGGTGATTACCCTGAAGTCCAAGCTGGTCAGCGATTTCAGAAAGGACTTTCAATTCTACAAAGTGCGGGAGATCAATAACTATCATCATGCTCATGACGCATATCTGAATGCCGTGGTGGGAACCGCCCTGATCAAGAAGTACCCAAAGCTGGAAAGCGAGTTCGTGTACGGAGACTACAAGGTCTACGACGTGCGCAAGATGATTGCCAAATCTGAGCAGGAGATCGGAAAGGCCACCGCAAAGTACTTCTTCTACAGCAACATCATGAATTTCTTCAAGACCGAAATCACCCTTGCAAACGGTGAGATCCGGAAGAGGCCGCTCATCGAGACTAATGGGGAGACTGGCGAAATCGTGTGGGACAAGGGCAGAGATTTCGCTACCGTGCGCAAAGTGCTTTCTATGCCTCAAGTGAACATCGTGAAGAAAACCGAGGTGCAAACCGGAGGCTTTTCTAAGGAATCAATCCTCCCCAAGCGCAACTCCGACAAGCTCATTGCAAGGAAGAAGGATTGGGACCCTAAGAAGTACGGCGGATTCGATTCACCAACTGTGGCTTATTCTGTCCTGGTCGTGGCTAAGGTGGAAAAAGGAAAGTCTAAGAAGCTCAAGAGCGTGAAGGAACTGCTGGGTATCACCATTATGGAGCGCAGCTCCTTCGAGAAGAACCCAATTGACTTTCTCGAAGCCAAAGGTTACAAGGAAGTCAAGAAGGACCTTATCATCAAGCTCCCAAAGTATAGCCTGTTCGAACTGGAGAATGGGCGGAAGCGGATGCTCGCCTCCGCTGGCGAACTTCAGAAGGGTAATGAGCTGGCTCTCCCCTCCAAGTACGTGAATTTCCTCTACCTTGCAAGCCATTACGAGAAGCTGAAGGGGAGCCCCGAGGACAACGAGCAAAAGCAACTGTTTGTGGAGCAGCATAAGCATTATCTGGACGAGATCATTGAGCAGATTTCCGAGTTTTCTAAACGCGTCATTCTCGCTGATGCCAACCTCGATAAAGTCCTTAGCGCATACAATAAGCACAGAGACAAACCAATTCGGGAGCAGGCTGAGAATATCATCCACCTGTTCACCCTCACCAATCTTGGTGCCCCTGCCGCATTCAAGTACTTCGACACCACCATCGACCGGAAACGCTATACCTCCACCAAAGAAGTGCTGGACGCCACCCTCATCCACCAGAGCATCACCGGACTTTACGAAACTCGGATTGACCTCTCACAGCTCGGAGGGGATGAGGGAGCTCCCAAGAAAAAGCGCAAGGTAGGTTAATGAgaattccagctgagcgccggtcgctaccattaccagttggtctggtgtcaaaaataataataaccgggcaggccatgtctgcccgtatttcgcgtaaggaaatccattatgtactatttaaaaaacacaaacttttggatgttcggtttattctttttcttttacttttttatcatgggagcctacttcccgtttttcccgatttggctacatgacatcaaccatatcagcaaaagtgatacgggtattatttttgccgctatttctctgttctcgctattattccaaccgctgtttggtctgctttctgacaaactcggaacttgtttattgcagcttataatggttacaaataaagcaatagcatcacaaatttcacaaataaagcatttttttcactgcattctagttgtggtttgtccaaactcatcaatgtatcttatcatgtctggatcgacaaagtcaaagcggccatcagatctgccggtctccctatagtgagtcgtattaatttcgataagccaggttaacctgcattaatgaatcggccaacgcgcggggagaggcggtttgcgtattgggcgctcttccgcttcctcgctcactgactcgctgcgctcggtcgttcggctgcggcgagcggtatcagctcactcaaaggcggtaatacggttatccacagaatcaggggataacgcaggaaagaacatgtgagcaaaaggccagcaaaaggccaggaaccgtaaaaaggccgcgttgctggcgtttttccataggctccgcccccctgacgagcatcacaaaaatcgacgctcaagtcagaggtggcgaaacccgacaggactataaagataccaggcgtttccccctggaagctccctcgtgcgctctcctgttccgaccctgccgcttaccggatacctgtccgcctttctcccttcgggaagcgtggcgctttctcaatgctcacgctgtaggtatctcagttcggtgtaggtcgttcgctccaagctgggctgtgtgcacgaaccccccgttcagcccgaccgctgcgccttatccggtaactatcgtcttgagtccaacccggtaagacacgacttatcgccactggcagcagccactggtaacaggattagcagagcgaggtatgtaggcggtgctacagagttcttgaagtggtggcctaactacggctacactagaaggacagtatttggtatctgcgctctgctgaagccagttaccttcggaaaaagagttggtagctcttgatccggcaaacaaaccaccgctggtagcggtggtttttttgtttgcaagcagcagattacgcgcagaaaaaaaggatctcaagaagatcctttgatcttttctacggggtctgacgctcagtggaacgaaaactcacgttaagggattttggtcatgagattatcaaaaaggatcttcacctagatccttttaaattaaaaatgaagttttaaatcaatctaaagtatatatgagtaaacttggtctgacagttaccaatgcttaatcagtgaggcacctatctcagcgatctgtctatttcgttcatccatagttgcctgactccccgtcgtgtagataactacgatacgggagggcttaccatctggccccagtgctgcaatgataccgcgagacccacgctcaccggctccagatttatcagcaataaaccagccagccggaagggccgagcgcagaagtggtcctgcaactttatccgcctccatccagtctattaattgttgccgggaagctagagtaagtagttcgccagttaatagtttgcgcaacgttgttgccattgctacaggcatcgtggtgtcacgctcgtcgtttggtatggcttcattcagctccggttcccaacgatcaaggcgagttacatgatcccccatgttgtgcaaaaaagcggttagctccttcggtcctccgatcgttgtcagaagtaagttggccgcagtgttatcactcatggttatggcagcactgcataattctcttactgtcatgccatccgtaagatgcttttctgtgactggtgagtactcaaccaagtcattctgagaatagtgtatgcggcgaccgagttgctcttgcccggcgtcaatacgggataataccgcgccacatagcagaactttaaaagtgctcatcattggaaaacgttcttcggggcgaaaactctcaaggatcttaccgctgttgagatccagttcgatgtaacccactcgtgcacccaactgatcttcagcatcttttactttcaccagcgtttctgggtgagcaaaaacaggaaggcaaaatgccgcaaaaaagggaataagggcgacacggaaatgttgaatactcatactcttcctttttcaatattattgaagcatttatcagggttattgtctcatgagcggatacatatttgaatgtatttagaaaaataaacaaataggggttccgcgcacatttccccgaaaagtgccacctgacgtctaagaaaccattattatcatgacattaacctataaaaataggcgtatcacgaggcccttacgtattaattaa

>H2B::mCherry H2B from *Helobdella* spp. leech mCherry

ATGCCACCAAAGCCTGCCAGCAAGGGAGCTAAGAAGGCCGCCAGCAAGGCGAAAGCTGCTCGCAGCACGGACAAGAAGCACAAGAGAAGGCGAAAGGAAAGCTACTTTATATACATATACAAAGTGCTGAAGCAGGTTCACCCGGACACGGGCATCAGCGGCAAAGCCATGTCAATAATGAACTCGTTCGTCAATGACATCTTTGAACGAATCGCAGCCGAAGCTTCTCGCCTCGCCCACTACAACAAGAGATCCACAATCACCAGCAGAGAAATCCAGACGGCCGTCAGGCTTCTGTTACCCGGCGAGTTGGCCAAGCACGCCGTCAGCGAAGGCACCAAGGCCGTCACCAAGTACACCAGCTCAAAGgtcgacaggccaatctggccgcgggtcgacggtaccgcgggcccgggatccatcgccaccATGGTGAGCAAGGGCGAGGAGGATAACATGGCCATCATCAAGGAGTTCATGCGCTTCAAGGTGCACATGGAGGGCTCCGTGAACGGCCACGAGTTCGAGATCGAGGGCGAGGGCGAGGGCCGCCCCTACGAGGGCACCCAGACCGCCAAGCTGAAGGTGACCAAGGGTGGCCCCCTGCCCTTCGCCTGGGACATCCTGTCCCCTCAGTTCATGTACGGCTCCAAGGCCTACGTGAAGCACCCCGCCGACATCCCCGACTACTTGAAGCTGTCCTTCCCCGAGGGCTTCAAGTGGGAGCGCGTGATGAACTTCGAGGACGGCGGCGTGGTGACCGTGACCCAGGACTCCTCCCTGCAGGACGGCGAGTTCATCTACAAGGTGAAGCTGCGCGGCACCAACTTCCCCTCCGACGGCCCCGTAATGCAGAAGAAGACCATGGGCTGGGAGGCCTCCTCCGAGCGGATGTACCCCGAGGACGGCGCCCTGAAGGGCGAGATCAAGCAGAGGCTGAAGCTGAAGGACGGCGGCCACTACGACGCTGAGGTCAAGACCACCTACAAGGCCAAGAAGCCCGTGCAGCTGCCCGGCGCCTACAACGTCAACATCAAGTTGGACATCACCTCCCACAACGAGGACTACACCATCGTGGAACAGTACGAACGCGCCGAGGGCCGCCACTCCACCGGCGGCATGGACGAGCTGTACAAGTAA

>U6>Ebf.C sgRNA U6 promoter Ebf.C unique sequence F+E scaffold

ggcgcgcctggcgggtgtattaaaccactaaacaaacaattgccccaagctctcttcacaattataaacactataatgtttggacaagagattagcgtggctgtgacgagaactctcaaaggcttggtgtaattgatattttataagaagcagattaaacttcaatacagtttacacctcatttacaaaaaattggctgccaaaatcgctaatttacacatatttaaaacaatttcaacacatatacacagtatagtataactgcataataaacaaatacatctaacagacactcactaatctgccataacaagcttcaaaaacttaaactcgaaattttagtgaatctttttttttaaatgaatattttatttaaaaagttaaaaatattacagttcaggtataggtttacacctaatctttaataatccgaactaaattttaactatttagaaactttttcaaccaaagtttaaaaaaatagatttttcgcacgctaaaactatcatttacacaaaaaaatgcaacaaaatgcagaaaaaaattacattagagtttaggttagttacctgctaatcaatataaactaacttcccgcataatattcatctaaaattagcaataatcacgttttacgctaaaatttgtgtaaaactaaacttcgtcctttgtcaaggagaaaatttgactcaaaagctgcgcgcgcaggggagatccccaagcgagtgtttgttacatcataatcatgtggaaaaatcccctaataagtaaaaatacatattttttaattttgggggcaaataaaccgctttttatgtctaaaaacgccaaaaatggatcgcgcgagcccaaaaacgcacaaataacgtacagacagtgtctctgcgtacacagacggtatttcccctttaaattgagaactagacttaagcacgcttataagtctggaaggcatccgatggtatagatgAGACTGTGCCAAGACACCCGTTTAAGAGCTATGCTGGAAACAGCATAGCAAGTTTAAATAAGGCTAGTCCGTTATCAACTTGAAAAAGTGGCACCGAGTCGGTGCTTTTTTTgaattc

>U6>control sgRNA U6 promoter control unique sequence F+E scaffold

ggcgcgcctggcgggtgtattaaaccactaaacaaacaattgccccaagctctcttcacaattataaacactataatgtttggacaagagattagcgtggctgtgacgagaactctcaaaggcttggtgtaattgatattttataagaagcagattaaacttcaatacagtttacacctcatttacaaaaaattggctgccaaaatcgctaatttacacatatttaaaacaatttcaacacatatacacagtatagtataactgcataataaacaaatacatctaacagacactcactaatctgccataacaagcttcaaaaacttaaactcgaaattttagtgaatctttttttttaaatgaatattttatttaaaaagttaaaaatattacagttcaggtataggtttacacctaatctttaataatccgaactaaattttaactatttagaaactttttcaaccaaagtttaaaaaaatagatttttcgcacgctaaaactatcatttacacaaaaaaatgcaacaaaatgcagaaaaaaattacattagagtttaggttagttacctgctaatcaatataaactaacttcccgcataatattcatctaaaattagcaataatcacgttttacgctaaaatttgtgtaaaactaaacttcgtcctttgtcaaggagaaaatttgactcaaaagctgcgcgcgcaggggagatccccaagcgagtgtttgttacatcataatcatgtggaaaaatcccctaataagtaaaaatacatattttttaattttgggggcaaataaaccgctttttatgtctaaaaacgccaaaaatggatcgcgcgagcccaaaaacgcacaaataacgtacagacagtgtctctgcgtacacagacggtatttcccctttaaattgagaactagacttaagcacgcttataagtctggaaggcatccgatggtatagatgCTTTGCTACGATCTACATTGTTTAAGAGCTATGCTGGAAACAGCATAGCAAGTTTAAATAAGGCTAGTCCGTTATCAACTTGAAAAAGTGGCACCGAGTCGGTGCTTTTTTTgaattc
